# Are the Dietary Guidelines for Meat, Fat, Fruit and Vegetable Consumption Appropriate for Environmental Sustainability? A Review of the Literature

**DOI:** 10.3390/nu6062251

**Published:** 2014-06-12

**Authors:** Christian John Reynolds, Jonathan David Buckley, Philip Weinstein, John Boland

**Affiliations:** 1 Centre for Industrial and Applied Mathematics, the Barbara Hardy Institute, University of South Australia, Mawson Lakes Boulevard, Mawson Lakes, SA 5095, Australia; E-Mail: john.boland@unisa.edu.au; 2 Integrated Sustainability Analysis, University of Sydney, Sydney, 2000, Australia; 3 Nutritional Physiology Research Centre, Sansom Institute for Health Research, University of South Australia, Mawson Lakes Boulevard, Mawson Lakes, SA 5095, Australia; E-Mail: jon.buckley@unisa.edu.au; 4 School of Pharmacy and Medical Sciences, Division of Health Sciences, University of South Australia, Mawson Lakes Boulevard, Mawson Lakes, SA 5095, Australia; E-Mail: phil.weinstein@unisa.edu.au

**Keywords:** environmental impact, diet, food, red meat, animal protein, fat, fruit, vegetables

## Abstract

This paper reviews the current literature around the environmental impacts of dietary recommendations. The focus of the review is on collating evidence relating to environmental impacts of the dietary advice found in the World Health Organisation guidelines, and environmental impact literature: reducing the consumption of fat, reducing the consumption of meat-based protein and animal-based foods, and increasing the consumption of fruit and vegetables. The environmental impact of reducing dietary fat intake is unclear, although reducing consumption of the food category of edible fats and oils appears to have little impact. However most, but not all, studies support environmental benefits of a reduced consumption of animal-based foods and increased consumption of fruit and vegetables. In general, it appears that adhering to dietary guidelines reduces impact on the environment, but further study is required to examine the environmental impacts of animal-based foods, and fruit and vegetable intake in depth.

## 1. Introduction

Food consumption contributes an estimated 20% to 30% of the total adverse environmental impact in the Western world [1,2], being linked to soil, air, and water pollution and loss of biodiversity [3,4,5,6,7,8,9,10,11,12,13,14,15,16]. Despite this having been recognised for some time, the idea of altering diet to increase environmental sustainability is a relatively new concept that until the last decade had little real-life implementation [3,4,17,18].

Currently, dietary guidelines in most jurisdictions are mainly used to promote healthy eating to prevent chronic disease [19,20], with environmental, economic, or social impacts of diet considered to be externalities. Typically, as seen in the global dietary guidelines discussed by the World Health Organization (WHO) and the United Nations Food and Agriculture Organization (FAO) [21], the dialogue on environmental benefits is tempered by the focus on health, and the need to provide practical advice that people can follow. Environmental considerations, if mentioned, are relegated to the appendices—as in the current Australian Dietary guidelines [22,23]. Rare exceptions to this trend are the recent publications by the Health Council of the Netherlands [24] and the Nordic Council of Ministers [25] which discuss a healthy diet from an ecological perspective.

Globally there is much debate over what constitutes a healthy diet, how to optimise diet, and how to present this information to the general population [26,27,28,29,30,31,32,33]. There are national and international “healthy” portion sizes, and recommended daily allowances of differing foodstuffs based upon caloric content, cultural, historic and economic factors. Yet many studies express difficulty in finding an individual eating in perfect concordance with global dietary guidelines [21,34,35,36].

Since the 1960s there has been a marked increase in the variety of dietary guidelines published [37,38,39]. Concurrently, the global diet has shifted due to the rising global average income, and greater access to cheap, highly processed foodstuffs and animal products, resulting in increasing rates of obesity and chronic disease [40]. The intensification of publication and debate over recommended diets can be seen, in part, as a reaction to this changing global diet and the adverse impacts on health. Yet, as indicated above, discussion around the environmental impacts of recommended dietary guidelines is now only emerging [41].

In this paper we focus on dietary guidelines, excluding nutrient guidelines (such as [42]). The sheer variety of foods that an individual can choose from to obtain the recommended daily nutrients, results in greater complexity with respect to the associated environmental impacts. Due to the complexity we believe that nutrient guidelines are deserving of their own analysis.

In this paper we review the current environmental impact assessment and life cycle analysis (LCA) literature around the environmental impacts of dietary recommendations, focusing on collating the environmental evidence behind three pieces of dietary advice that are debated in current environmental impact assessment and LCA literature, and also presented in the WHO guidelines: reducing the consumption of fat, reducing the consumption of meat-based protein and animal-based foods, and increasing the consumption of fruit and vegetables [21,32,33]. Environmental impacts of dietary advice presented in WHO guidelines to reduce sugar consumption have not been evaluated in the LCA literature and was therefore unable to be included in this review. The lack of assessment of the impact of this guideline indicates a gap in the literature that should be addressed in future studies.

## 2. Reducing Fats

Since the 1960s the average global daily consumption of fat has increased by 20 g per person (27%) [21]. This rise in fat consumption is proposed to be due to an increase in the availability and consumption of cheaper energy-dense, high-fat, nutrients-poor food stuffs [43,44,45,46] such as processed snacks, caloric beverages, fast foods, and edible oils and spreadable fats. The increase in fat intake, and associated increase in energy intake, has been blamed for contributing to an epidemic increase in overweight and obesity and associated health conditions, with 1.5 billion people classified as overweight and over 500 million as obese [40,47].

Fats can be understood to be an independent category of food, as well as a nutritional component of a broad range of foodstuffs. Thus, fats can be directly consumed as edible oils and spreads, or indirectly consumed in food sources such as dairy, meat, *etc*. This dual nature of the dietary availability of fats has meant that dietary and nutrient advice has overlapped in discussing healthy fat consumption levels. As a result most dietary guidelines provide recommendations for total fat consumption, rather than specific recommendations for consumption of fat as edible oils and spreadable fats. The current WHO guidelines recommend that 15%–30% of dietary energy be supplied from fats. However, the actual amount of dietary energy derived from fats is country dependent, with the figure for developed nations being around 20%–40% [21,48,49]. This recommendation by the WHO to limit fat consumption is based on recommendations aimed at improving health rather than environmental impacts, and relates to total intake of fat from all sources, including not only foods from the food category of edible fats and oils, but also fat contained as part of the nutrient profile of other foods. In terms of environmental impacts however, because of the assessment methodologies used, it has to date only been possible to estimate the environmental impacts of edible fats and oils as a food category and not to evaluate the independent environmental effects of fats that form part of the composition of other food categories. For example, meat contains fat as part of its nutritional profile, but separate analysis cannot currently be undertaken to estimate the environmental impact of the fat content of the meat independently from the protein and other nutrients as the current methodologies available do not permit this level of analysis. Thus, at present analysis of the environmental impacts of fat is limited to analysis of the food category of edible fats and oils.

Vieux *et al.* [50] examined the greenhouse gas effects of reducing the consumption of energy-dense, high-fat, nutirent-poor food stuffs, and found that the food category of edible fats contributed 7% of daily diet-associated greenhouse gas emissions compared to fruit and vegetables at 9%, or meat at 27%. Considering that the French diet sources over 40% of its energy from fat-type products [49], it can be understood that edible fats do not have a large (nor proportional) environmental impact when compared to fruit, vegetables or meat products. In a subsequent study by these same authors, which also evaluated total dietary fat intake (*i.e.*, nutrient analysis), it was reported that while total dietary fat intake was higher in lower nutritional quality diets, these diets were associated with lower greenhouse gas emissions [51], but this latter study was unable to identify the specific contribution of dietary fat to greenhouse gas emissions and the lower greenhouse gas emissions may have been related to nutrients other than fat. One strength of these studies was that they evaluated self-selected diets from a random sample of the French population, and therefore reflect the environmental impacts of the actual diversity of food consumption patterns, but a limitation was that they examined the environmental impacts of different food categories rather than the impacts of individual dietary nutrients. Additional research should examine the proportion of environmental consequences from the intake of indirect fats (as nutrients) as opposed to the food category of fats and oils.

Despite the apparently low environmental impact of fat consumption, it has been proposed that fat intake be reduced to improve health, with a possible mechanism to facilitate this being the introduction of a “fat” tax. However, it has been proposed that such a tax would represent an economic device to raise revenue rather than alter diet [43,45,46,52,53,54,55,56,57]. Conversely, it has been further suggested that fat taxes could be used to increase prices to reflect the actual social cost of food, including the cost of ameliorating environmental impacts [58,59,60,61]. A common criticism of “fat” taxes is that they are regressive—with low-income households being forced pay a greater percentage of their income than higher income households. Furthermore, the environmental impacts of a fat tax have not been well examined, with only Friel *et al.* [62] providing some discussion on the merits of fat taxes to reduce consumption of GHG intensive goods. In particular, Friel *et al.* [62] discussed that fat-taxes—though useful—should only be part of a behaviour change tool set, and could also be used to “link health and climate-change agendas”.

Thus, the reduction of fat consumption via price mechanisms may produce some monetary and health benefits. However, the environmental impact of a reduction in fat consumption is unclear as methods are only available to model the impact of reduced intake of edible fats and oils as a food category and not the impacts of fats that form a nutritional component of other food groups. However, a reduction of fat consumption via the eating of low fat (or fat removed) foods could result in the removed fat becoming food waste if not used by other industries [63,64,65]. In turn this waste could produce problematic environmental consequences. Further study of the indirect environmental impacts of a low fat diet is required.

## 3. Reducing Meat and Animal-Based Foods

Since the 1960s, the consumption of animal-based foods has risen throughout the world at the expense of consumption of non-animal-based staple foods such as grains, pulses, and fruits and vegetables [6]. This is due to increased production efficiency of the meat and dairy industries [66,67,68,69,70], higher standards of living and a rising global average income with an increasing demand for meat [71,72,73]. This is most evident in China with total meat consumption increasing 165% since 1990, while in Asia as a whole it has increased 30-fold since 1961 [74]. However, there is some evidence that meat consumption in Asia may have peaked, and that these countries may now not be following the developed world’s consumption pattern for more meat [71,73,74].

The consumption of meat and animal products offers essential (micro) nutritional security to many who would be otherwise food insecure [75,76,77]. However, excessive consumption of meat and animal products in some countries, and in some social classes within countries, can lead to excessive intakes of fat (nutrients), which can impact adversely on health [78]. This has led to the recommendation in some dietary guidelines [22] to limit meat consumption, in particular processed meat and, for men to reduce their intake of red meat. Even with such recommendations, the FAO is projecting a global yearly consumption of 45 kg of meat and 95 kg of dairy per person by the year 2030 [21]. Though this is below the 1997/9 average yearly meat and milk consumption of both industrialised (88 kg, 212 kg) and transition (46 kg, 159 kg) economies [71], is still above levels that many consider to be sustainable [74,79,80].

The environmental impact of meat and animal product consumption has been the topic of some investigation [6,67,68,76,79,80,81,82,83,84,85,86]. It has been found that meat-centric meals generate on average nine times higher greenhouse gas emissions than plant-based equivalents [5], while specific meat-based products such as beef and cheese cause 10–20 times more environmental impact [87,88,89]. An animal-based diet requires 2.5–5.0 times the energy inputs [7,90,91,92,93], 2–3 times the water, 13 times the fertilizer, and 1.4 times the pesticide use per calorie produced compared with a plant-based diet [7,93,94]. In European life cycle assessment studies, because of the relatively high meat intake in the typical diet, meat-free scenarios were between 18% and 31% lower in greenhouse gas emissions than the average diet [17,41].

Though there is literature suggesting an environmentally friendly diet can be achieved with meat and dairy products present [28], there have been many arguments mounted against meat dominant diets [5,93,95,96,97,98]. If animal-based foods are to be part of the diet, the selection of the least environmentally damaging foods is crucial. McMichael *et al.* [98] modelled a working global yearly meat intake target of no more than 32 kg per person with no more than 18 kg per year coming from red meat from ruminants (*i.e.*, cattle, sheep, goats, *etc.*). This is below the projected intake of 45 kg of meat for a meat-reduced diet cited in the aforementioned WHO/FAO meat consumption projections.

Besides selecting non-ruminant animals, another way to minimise the impact of animal products is to use farming practices that are suitable to the land type, and select less environmentally damaging feed and fodder [17]. These farming practices (lot-fed compared to grass-fed) can result in pronounced differences of environmental impacts. Studies [87,99,100] have shown this variance to be dependent on the type and geography of the farmed land and the differences can be minimal [101]—grass feeding having a potentially greater environmental impact than lot feeding in arid areas, while in developed nations with temperate climates, lot feeding can have greater impacts depending on the production systems [86].

The larger contribution to the environmental impact of animals that can be altered is the feed used [102,103,104]. Currently there is a large dependency on cereals and legumes (such as wheat, corn and soy) for animal feed with 37% of global cereal production being fed to animals [76]. Traditionally farming of these cereals has been resource intensive with a sizeable environmental footprint [105,106]. Regardless of the sustainable intensification techniques that are now being implemented, demand for these cereals as animal feed (along with bio fuel production) is currently growing, resulting in global deforestation and biodiversity threats [8]. Switching to alternative sources of animal feed that are less resource intensive might therefore provide a viable method for reducing the environmental impact of animal-based foods. More potentially sustainable alternatives include by-products from other agricultural sectors (such as molasses cake, brewers’ grains, vegetable residues and rice husks) [76]. The current level of production from by-product feedstock is limited, despite Fadel [107] finding that there was theoretically enough nutritional content available to provide production for 80% of global milk consumption in 1993. However, this study included processed soymeal as a feed, which Garnett [76] indicates is not a by-product *per se*, nor can soy meal be claimed to be resource efficient, because industrial soy farming can have many negative environmental consequences [104,108,109].

Fish and seafood consumption provides animal protein from a non-red meat source. Currently, fish and seafood provides more than 4 billion people with approximately 15% of their intake of animal protein, which equates to a global yearly consumption of approximately 18.6 kg of fish per person [21,110]. This consumption of fish is above the level of population growth, with 57% of global fish stocks now fully exploited (*i.e.*, at or very close to their maximum sustainable production) and 30% overexploited [110]. To meet demand and to combat the problems of over fishing wild-caught fish stocks there has been a marked increase in global aquaculture (with much occurring in Asia and Sub-Saharan Africa [111,112,113,114,115]), and a recent World Bank report [116] stated that by 2030, aquaculture will provide close to two thirds of global food fish consumption (186 million tons). With the increasing use of aquaculture, Merino *et al.* [117] have determined that the fish demand by 2050 will be met, but only if fish resources are managed sustainably and the animal feeds industry reduces its reliance on wild fish. While increasing aquaculture may assist in preventing depletion of wild fish stocks, both wild-caught and aquafarmed fish have substantial environmental impacts, with fish protein being up to 14 times more energy-intensive to produce than a vegetable equivalent [93].

From these statistics, the case can easily be made that reducing the intake of animal protein (including from fish) and dairy foods in the global diet would potentially have considerable impact on reducing environmental effects. However, this is likely to be unpalatable to much of the global population for many cultural, nutritional, and economic reasons [28,118]. Nevertheless, from an environmental perspective the dietary advice to reduce animal based foods is most welcome.

## 4. Increasing Fruit and Vegetable Intake

Fruits and vegetables play a key role in providing a diverse and nutritious diet, with studies showing that adequate consumption of fruits and vegetables reduces the risk of chronic disease [119,120,121]. However, unlike red meat, the global consumption of fruits and vegetables has persistently been below recommended guidelines [122], with over 77% of men and women in low- and middle-income countries consuming less than the WHO’s minimum recommended 400 g per capita per day of fruits and vegetables. Consumption of fruits and vegetables for many high income countries is also lower than the WHO’s minimum recommended volumes [21,33,34,35,123].

The environmental impact of fruits and vegetables varies greatly according to the individual type and production method [124,125,126,127,128]. Thus, it is more useful to contrast typical diets with diets high in vegetables and fruit, or high in animal-based foods [5,98,129,130]. In these dietary comparisons it has been found that greenhouse gas emissions with diets high in vegetables and fruit are lower than typical diets or diets high in animal-based foods. A number of studies have also reported that vegetarian diets are more environmentally friendly than other dietary patterns [7,93,94,131]. Furthermore, Baumann [132] found that vegan diets produced 23% less greenhouse gas emissions than the average vegetarian diet. However, it should be noted that from a food security and diet perspective, vegan and vegetarian diets, though lower in environmental impacts, have nutritional risks [133]. Specifically, there is greater potential for the insufficient intake of certain micronutrients [41,88,134]. This matters most when diet is of a monotonous limited selection, when even a small animal-based food intake could make a critical difference to micronutrient intake [75,76,77]. Thus the advice for a diet high in fruit and vegetables, with some meat products, has some merit from the food security viewpoint as well as opposed to vegetarian or vegan diets.

While the majority of evidence suggests that an increased intake of fruit and vegetables will reduce environmental impact, there is a small (but growing) literature that suggests a diet low in meat and high in fruits and vegetables is not always low in environmental impact [135]. This is because, in some cases, the quantity of vegetable substitutes eaten to replace animal proteins can contribute similar levels of environmental impacts [50,51], due to the increased quantities of cereals and vegetables for human consumption only slightly outweighing the corresponding decline in the land, water, and resources required to grow feed-cereal previously destined for animals [49]. There needs to be additional modelling to test these claims.

## 5. Conclusions

There are a myriad of possibly sustainable diets, with the components of each part of a diet contributing different volumes of environmental impacts [28]. In this paper we have examined three of the most common pieces of advice found in dietary guidelines. We found evidence of environmental benefits from reducing animal product intake and increasing fruit and vegetable consumption. However, there is also a small (but growing) section of the literature that suggests a diet low in meat and high in fruits and vegetables is not always the most environmentally friendly [135]. Further study is required to examine the veracity and suitability of these claims for the various global diets.

We found little research into the environmental impact of reducing fat in the diet. The most recent study to examine the environmental impact of direct edible fats found that fat currently accounts for less GHG emissions than vegetables, while contributing a larger share of dietary energy. This finding gives weight to the argument that the diets lowest in GHG emissions may not be lowest in fat [51]. Further investigation is needed into the environmental impacts of both direct and indirect fats within contemporary global diets.

Importantly, there is clear evidence that the majority of the global population does not adhere to dietary advice. Our review suggests that further investigation into the environmental benefits of following dietary guidelines in comparison to contemporary reported dietary habits is required. Such evidence would give more strength to the argument for adopting recommended dietary guidelines.
